# Prevalence of Vitamin D Deficiency in Orthopedic Trauma Patients: A Cross-Sectional Survey From a Tertiary Care Trauma Center

**DOI:** 10.7759/cureus.69174

**Published:** 2024-09-11

**Authors:** Shailendra Singh, Yuvraj Vimal, Shubham Srivastava, Ravindra Mohan, Deepak Kumar, Devarshi Rastogi, Pranjal Gupta, Balwinder Singh, Anuradha Gupta

**Affiliations:** 1 Department of Orthopaedic Surgery, King George’s Medical University, Lucknow, IND; 2 Department of Orthopaedic Surgery, Sarojini Naidu Medical College, Agra, IND; 3 Department of Orthopaedic Surgery, Prasad Institute of Medical Sciences, Lucknow, IND; 4 Department of Orthopaedic Surgery, Rani Durgawati Medical College, Banda, IND; 5 Department of Pathology, Sarojini Naidu Medical College, Agra, IND

**Keywords:** fracture healing factors, fragility fractures, trauma and orthopedic surgery, vitamin d deficiency, vitamin d supplementation

## Abstract

Background

Vitamin D deficiency is prevalent globally, with potential consequences for bone health and trauma outcomes. This study aimed to assess the prevalence of vitamin D deficiency in orthopedic trauma patients and investigate its correlation with various demographic and injury-related factors.

Methodology

A cross-sectional investigation was undertaken at a tertiary care center. An evaluation of serum 25-hydroxyvitamin D3 levels was conducted on 124 individuals, aged 20 to 70 years, who were hospitalized with orthopedic injuries. Demographic information, the injury method, the bone involvement pattern, and socioeconomic status were documented. Statistical analysis was employed to evaluate the correlations between vitamin levels D and these variables.

Results

The overall prevalence of vitamin D deficiency was 54 (43.6%) cases, with nine (7.3%) cases exhibiting severe deficiency and 45 (36.3%) cases exhibiting moderate deficiency. Higher rates of deficiency were associated with lower socioeconomic status (p = 0.044) and low-velocity trauma (p = 0.037). No significant association was found with age, sex, or residence. Interestingly, patients with multiple fractures were more prone to deficiency compared to those with single fractures.

Conclusions

This survey revealed a significant vitamin D deficiency among orthopedic trauma patients. Factors such as socioeconomic status and the nature of the injury emerged as significant risk factors. While conducting routine vitamin D assessments might pose challenges in developing nations, consistent supplementation could prove advantageous in enhancing fracture healing and overall health outcomes among this demographic. There is a call for future research to delve deeper into the role of vitamin D in trauma management and refine supplementation strategies.

## Introduction

Vitamin D, a vital fat-soluble vitamin, plays a crucial role in human physiology. Sun exposure and diet (fortified foods, fish liver oils, and supplements are the primary sources of vitamin D; vegetables and grains are poor sources) account for most of its intake in humans. Vitamin D deficiency can affect individuals of all ages, genders, races, and geographical locations. Vitamin D deficiency has multiple consequences that are still being explored, aside from the well-known skeletal complications. Numerous studies have suggested that vitamin D insufficiency is associated with several pathological conditions, which has led to a great deal of scientific research on vitamin D in recent years [1,2]. 7-dehydrocholesterol, which is found in the skin, must be exposed to sunlight for it to be converted to vitamin D3. Afterward, it undergoes conversion into 25-hydroxyvitamin D3 in the liver, with further conversion occurring in the kidney to produce 1,25-dihydroxyvitamin D3, also known as calcitriol [2]. Calcitriol is the active form of vitamin D, and it binds to the receptor of vitamin D, thereby regulating mineral ion homeostasis through the modulation of gene transcription.

Vitamin D is regarded as essential for musculoskeletal health. Its primary function is to regulate calcium and phosphate balance, which ensures proper bone metabolism by maintaining an equilibrium between bone resorption and formation. The crucial role of vitamin D in the metabolism of calcium and maintaining bone health is thoroughly understood and established. Vitamin D deficiency has been shown to hinder fracture healing, potentially leading to the development of fracture nonunion [3]. Even though there are debates about the true frequency of hypovitaminosis, the inadequacy of vitamin D is likely more prevalent in developing countries across various age groups [4]. The burden and morbidity of skeletal trauma add to this deficiency and may adversely affect those who are injured [5,6]. Research on vitamin D levels in individuals with orthopedic injuries has unveiled notable instances of insufficiency, prompting evaluation and recommendations for supplementation [7,8].

The main source of vitamin D in Western countries is fortified food products [9]. Many developing countries have yet to implement policies on vitamin D fortification of foods, and the actual extent of deficiency is unknown. Limited research within specific population groups suggests that the prevalence of vitamin D deficiency in India is much higher than anticipated. This study aimed to ascertain the prevalence of vitamin D deficiency in individuals experiencing orthopedic injuries and establish the correlation of vitamin D deficiency with various demographic parameters such as age, sex, residence, religion, occupation, educational status, marital status, economic status, mode of injury, the pattern of bone involvement, and type of fracture (open fracture vs. closed fracture). We hypothesize that patients with low socioeconomic status and those with low-velocity trauma will have a higher prevalence of vitamin D deficiency. There is limited knowledge regarding the prevalence of vitamin D deficiency in the Indian population and its association with various parameters mentioned above.

## Materials and methods

All patients admitted to the Ortho Emergency Unit, Trauma Center, at our tertiary care hospital, between the ages of 20 and 70 years, were assessed for 25-hydroxyvitamin D3 levels. These levels were measured using the electrochemiluminescence immune assay system in the clinical biochemistry laboratory of our hospital. The criteria for categorizing patients were as follows: severely deficient (<10 ng/mL), moderately deficient (11-20 ng/mL), insufficient (21-32 ng/mL), or adequate/normal (>32 ng/mL) [7].

We excluded elderly patients, as many of these patients would be on supplements. Patients with deranged renal function tests were also excluded, as this can affect calcium metabolism and vitamin D levels. Demographic data, including patient’s name, age, sex, residence, religion, occupation, educational status, marital status, economic status, mode of injury, the pattern of bone involvement, and type of fracture (open fracture vs. closed fracture) were noted.

The Modified B.G. Prasad classification was used to determine the economic status of the patients (Table 1) [10].

**Table 1:** Proposed Modified B.G. Prasad’s social classification. SES: socioeconomic status

Prasad’s social classification (1961)		Revision of Prasad’s social classification (2020)	
Social class (SES)	Per-capita monthly income limits	Social class (SES)	Revised for 2020 (in INR/month)
I	100 and above	I	7,533 and above
II	50–99	II	3,766–7,532
III	30–49	III	2,260–3,765
IV	15–29	IV	1,130–2,259
V	Below 15	V	1,129 and below

In this study, fractures were categorized as high velocity if they were caused by falls from heights greater than one story or by high-speed motor vehicle collisions. Low-velocity fractures referred to those resulting from assaults with a blunt object, a closed fist, or falls from standing height [11]. A sample size of 124 was deemed necessary to assess the prevalence of vitamin D deficiency in individuals with orthopedic trauma, with a type I alpha error of 5%, ensuring a 95% confidence level, and a type beta II error of 10%, providing the study 90% power to detect significant results. A p-value below 0.05 was considered to be an indicator of statistical significance. Demographic information and details regarding the orthopedic injury were meticulously recorded for each patient. The analysis was conducted using version 16.0 of the SPSS software (SPSS Inc., Chicago, IL, USA). The outcomes were presented as both means and percentages.

## Results

The mean age of the patients was 35.34 ± 13.51 years, ranging from 20 to 70 years. The majority were in the 20-29-year age group, accounting for 52 (41.9%) cases, followed by the 30-39-year age group, accounting for 31 (25%) cases. Detailed demographic information is provided in Table 2.

**Table 2:** Distribution of cases according to age.

Age (years)	No.	%
20–29	52	41.9
30–39	31	25.0
40–49r	20	16.1
50–59	12	9.7
≥60	9	7.3
Total	124	100.0

**Figure 1:**
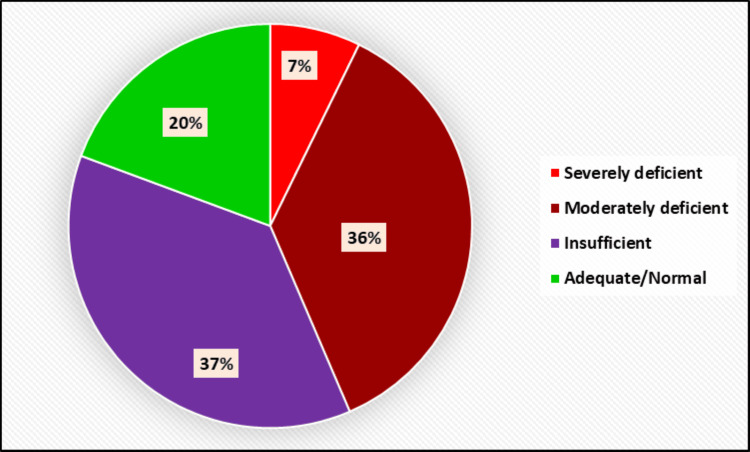
Distribution of vitamin D levels.

There were 83 (66.9%) males and 41 (33.1%) females among the 124 patients with orthopedic injuries who were admitted and treated in the orthopedic emergency unit at the Trauma Center. Severe vitamin D deficiency was observed in nine (7.3%) patients, while moderate deficiency was seen in 45 (36.3%) patients. Overall, 54 (43.6%) patients had some level of vitamin D deficiency. Additionally, 46 (37.0%) patients were found to be vitamin D insufficient, while 24 (19.4%) patients had normal vitamin D levels (Figure 1, Table 3).

**Table 3:** Distribution of vitamin D levels.

Vitamin D level	No.	%
Severely deficient	9	7.3
Moderately deficient	45	36.3
Insufficient	46	37.0
Adequate/Normal	24	19.4
Total	124	100.0

**Figure 2:**
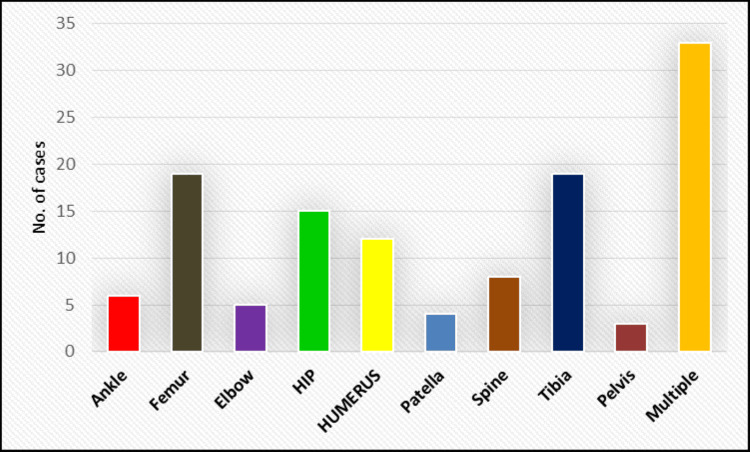
Association of vitamin D levels with the pattern of bone involvement.

The mode of injury was significantly associated with vitamin D deficiency (p = 0.037), with a higher rate of deficiency observed in low-velocity trauma cases (eight cases, 66.7%) (Table 4). The pattern of bone involvement also showed a significant link to vitamin D deficiency (p = 0.007), with severe deficiency found in cases with multiple fractures (five cases, 15.2%), hip fractures (three cases, 20%), and spine fractures (one case, 12.5%) (Figure 2, Table 5). Furthermore, economic status correlated significantly with vitamin D deficiency (p = 0.044), with the highest deficiency rate in SES V (six cases, 75%) followed by SES II (five cases, 62.5%) (Table 6).

**Table 4:** Association of vitamin D levels with the mode of injury.

Mode of injury	Vitamin D level								Chi-square	P-value
	Severely deficient (N = 9)		Moderately deficient (N = 45)		Insufficient (N = 46)		Adequate/ Normal (N = 24)			
	N	%	N	%	N	%	N	%		
High-velocity trauma	6	5.4%	40	35.7%	42	37.5%	24	21.4%	8.49	0.037
Low-velocity trauma	3	25.0%	5	41.7%	4	33.3%	0	0.0%		

**Table 5:** Association of vitamin D levels with the pattern of bone involvement.

Pattern of bone involvement	Vitamin D level								Chi-square	P-value
	Severely deficient (N = 9)		Moderately deficient (N = 45)		Insufficient (N = 46)		Adequate/Normal (N = 24)			
	N	%	N	%	N	%	N	%		
Ankle	0	0.0%	1	16.7%	5	83.3%	0	0.0%	48.10	0.007
Femur	0	0.0%	6	31.6%	8	42.1%	5	26.3%		
Elbow	0	0.0%	1	20.0%	0	0.0%	4	80.0%		
Hip	3	20.0%	6	40.0%	6	40.0%	0	0.0%		
Humerus	0	0.0%	3	25.0%	5	41.7%	4	33.3%		
Patella	0	0.0%	1	25.0%	2	50.0%	1	25.0%		
Spine	1	12.5%	1	12.5%	2	25.0%	4	50.0%		
Tibia	0	0.0%	11	57.9%	4	21.1%	4	21.1%		
Pelvis	0	0.0%	1	33.3%	2	66.7%	0	0.0%		
Multiple	5	15.2%	14	42.4%	12	36.4%	2	6.1%		

**Table 6:** Association of vitamin D levels with economic status.

Economic status	Vitamin D level								Chi-square	P-value
	Severely deficient (N = 9)		Moderately deficient (N = 45)		Insufficient (N = 46)		Adequate/Normal (N = 24)			
	N	%	N	%	N	%	N	%		
II	1	12.5%	4	50.0%	2	25.0%	1	12.5%	17.35	0.044
III	2	4.9%	12	29.3%	17	41.5%	10	24.4%		
IV	3	4.5%	26	38.8%	27	40.3%	11	16.4%		
V	3	37.5%	3	37.5%	0	0.0%	2	25.0%		

## Discussion

In our study, only 24 (19.4%) of the 124 patients had sufficient levels of vitamin D (>32 ng/mL). Cherian et al. [12] found that among 160 patients, only eight (5%) cases had adequate vitamin D levels. They reported a consistent prevalence of vitamin D deficiency, with 131 (82%) cases below 20 ng/mL, increasing to 152 (95%) cases below 32 ng/mL. Similarly, Smith et al. [13] observed that out of 75 patients, 35 (47%) had insufficient vitamin D levels, and 10 (13%) patients were deficient. Similarly, Aparna et al. [14] reported a prevalence of vitamin D deficiency ranging from 50% to 94% in their study. In most of these studies, vitamin D deficiency was defined as a serum 25-hydroxyvitamin D level of less than 20 ng/mL. Like our study, all these studies demonstrate an alarmingly high prevalence of vitamin D deficiency. This may be due to lifestyle factors such as reduced outdoor activities, a higher rate of obesity, and the significant number of vegetarians in this area.

In our study, we found a significant correlation between socioeconomic status and vitamin D deficiency (p = 0.044). Deficiency rates were highest in SES V, affecting six (75%) cases, with SES II next at five (62.5%) cases. Lin et al. [15] concluded in their study that women of childbearing age in rural northern China with lower socioeconomic status were at a higher risk of vitamin D deficiency or insufficiency. Similarly, Léger-Guist'hau et al. [16] found in their study that low socioeconomic status is an independent risk factor for vitamin D deficiency. This deficiency in patients with lower socioeconomic status may result from reduced access to vitamin D-rich foods such as fatty fish, fortified dairy products, and eggs due to their higher cost. Additionally, they often face limited access to healthcare services, including routine check-ups and preventive care, as well as a lack of awareness.

Our study also revealed a significant association between the mode of injury and vitamin D deficiency (p = 0.037), with a higher rate of deficiency observed in individuals with low-velocity trauma, affecting eight (66.7%) cases. Smith et al. [13] conducted a study and concluded that vitamin D deficiency was highly prevalent in patients with low-energy fractures, particularly among those who were smokers, obese, or had other medical risk factors for vitamin D deficiency. Konstantinidis et al. [17] showed that elderly patients with low-energy hip fractures living in a sunny Mediterranean region still exhibited poor vitamin D levels. These findings confirm our initial hypothesis. As vitamin D plays a crucial role in bone mineralization, insufficient levels can lead to an increased risk of fractures.

Additionally, our study identified a significant association between the pattern of bone involvement and vitamin D deficiency (p = 0.007). Severe deficiency was notably prevalent in cases with multiple fractures (five cases, 15.2%), hip fractures (three cases, 20%), and spine fractures (one case, 12.5%). However, Ribbans et al. [18] found no significant link between vitamin D levels and the anatomical location of the injury in their study. In a literature review by Maier et al. [3], it was found that numerous studies reported a high incidence of vitamin D deficiency in women with hip and vertebral fractures. The pattern of bone involvement in vitamin D deficiency is characterized by generalized bone weakening, with a higher incidence of fractures in specific, high-stress areas such as the hips and spine.

In 2021, Moon et al. [19] published a case report on the importance of vitamin D in the management of fracture non-union. A 44-year-old healthy male presented with complaints of pain due to the non-union of a femoral shaft fracture, four years after the initial trauma. Despite three previous operative interventions with proper anatomical fixation, the fracture did not unite. Further investigations revealed a vitamin D deficiency, and after supplementation with vitamin D, a complete union of the fracture was noticed. This case beautifully demonstrates that an adequate serum level of vitamin D is a prerequisite for fracture healing. With the rising prevalence of deficiency of vitamin D and associated complications in fractures, it is recommended to routinely assess vitamin D levels as part of the clinical evaluation in cases of fracture non-union.

Our study’s findings are consistent with previous research that has identified a high prevalence of vitamin D deficiency among patients with fractures. However, our focus on a population from a tertiary care center in a developing country adds a unique perspective to the global understanding of this issue. Vitamin D plays a critical role in bone metabolism and immune function, both of which are crucial for fracture healing [20,21]. The deficiency observed in our cohort may contribute to delayed union or non-union of fractures, potentially leading to prolonged recovery times and increased healthcare costs.

The study’s single-center design and limited sample size may impact the broader applicability of its findings, although the results are consistent with previous research. The absence of multivariate analysis and the exclusion of confounding factors such as sun exposure, clothing habits, and dietary intake might affect the interpretation of the data. Some research indicates that vitamin D deficiency could negatively impact bone healing, potentially resulting in delayed or incomplete union. Future studies should be larger, multi-center, randomized controlled trials that utilize multivariate analysis. These trials could involve fracture patients receiving varying vitamin D doses and assess their healing progress periodically using established scoring systems to gain a deeper understanding of the role of vitamin D role in fracture healing.

## Conclusions

Our study highlights several important insights into vitamin D deficiency among patients with orthopedic injuries. The findings reveal a significant association between socioeconomic status and vitamin D deficiency, with the highest deficiency rates observed in individuals from lower socioeconomic backgrounds. Additionally, our results indicate a notable correlation between the mode of injury and vitamin D deficiency, particularly in cases of low-velocity trauma. The pattern of bone involvement also emerged as a significant factor, with severe deficiency prevalent in patients with multiple fractures, hip fractures, and spine fractures. While routine vitamin D level assessments for all trauma center admissions may not be feasible in a developing country such as India, regular vitamin D supplementation can yield positive results for trauma patients and reduce the risk of fractures with low-velocity trauma.

These findings underscore the need for targeted interventions to address vitamin D deficiency, particularly among high-risk groups such as those with lower socioeconomic status and specific injury patterns. Future research should focus on exploring effective strategies for vitamin D supplementation and prevention, as well as examining the broader implications of vitamin D deficiency on recovery outcomes in orthopedic trauma patients. By addressing these issues, we can improve patient outcomes and contribute to more effective management of vitamin D deficiency in clinical settings.

## References

[1] Mithal A, Wahl DA, Bonjour JP (2009). Global vitamin D status and determinants of hypovitaminosis D. Osteoporos Int.

[2] Holick MF (2007). Vitamin D deficiency. N Engl J Med.

[3] Maier GS, Weissenberger M, Rudert M, Roth KE, Horas K (2021). The role of vitamin D and vitamin D deficiency in orthopaedics and traumatology-a narrative overview of the literature. Ann Transl Med.

[4] Suresh Kumar R, Syed S, Anand Kumar A, Subha Kumari KN, Sajitha K (2013). Serum vitamin D levels in Indian patients with multiple sclerosis. Indian J Clin Biochem.

[5] Burckhardt P (2012). [Calcium and Vitamin D in the treatment and prevention of osteoporosis: the actual dilemma]. Ther Umsch.

[6] Spedding S, Vanlint S, Morris H, Scragg R (2013). Does vitamin D sufficiency equate to a single serum 25-hydroxyvitamin D level or are different levels required for non-skeletal diseases?. Nutrients.

[7] Bee CR, Sheerin DV, Wuest TK, Fitzpatrick DC (2013). Serum vitamin D levels in orthopaedic trauma patients living in the northwestern United States. J Orthop Trauma.

[8] van den Bergh J, van Geel T, Geusens P (2010). [Should the vitamin D level be determined for all fracture patients?]. Ned Tijdschr Geneeskd.

[9] Yetley EA (2008). Assessing the vitamin D status of the US population. Am J Clin Nutr.

[10] Debnath DJ, Kakkar R (2020). Modified BG Prasad Socio-economic Classification, Updated - 2020. Indian J Comm Health.

[11] Roumeliotis G, Ahluwalia R, Jenkyn T, Yazdani A (2015). The Le Fort system revisited: trauma velocity predicts the path of Le Fort I fractures through the lateral buttress. Plast Surg (Oakv).

[12] Cherian VM, Gouse M, Albert S, Jayasankar V (2015). Prevalence of vitamin D deficiency in patients presenting with an orthopaedic trauma at a tertiary centre in South India - implications and protocols for replacement therapy. Malays Orthop J.

[13] Smith JT, Halim K, Palms DA, Okike K, Bluman EM, Chiodo CP (2014). Prevalence of vitamin D deficiency in patients with foot and ankle injuries. Foot Ankle Int.

[14] Aparna P, Muthathal S, Nongkynrih B, Gupta SK (2018). Vitamin D deficiency in India. J Family Med Prim Care.

[15] Lin S, Jiang L, Zhang Y, Chai J, Li J, Song X, Pei L (2021). Socioeconomic status and vitamin D deficiency among women of childbearing age: a population-based, case-control study in rural northern China. BMJ Open.

[16] Léger-Guist'hau J, Domingues-Faria C, Miolanne M (2017). Low socio-economic status is a newly identified independent risk factor for poor vitamin D status in severely obese adults. J Hum Nutr Diet.

[17] Konstantinidis C, Psoma O, Kotsias C (2024). Vitamin D deficiency in patients with low-energy hip fractures in accordance with the Mediterranean paradox. Cureus.

[18] Ribbans WJ, Aujla RS, Ashour R, Allen PE, Wood EV (2019). Vitamin D and foot and ankle trauma: an individual or societal problem. Foot (Edinb).

[19] Moon AS, Boudreau S, Mussell E, He JK, Brabston EW, Ponce BA, Momaya AM (2019). Current concepts in vitamin D and orthopaedic surgery. Orthop Traumatol Surg Res.

[20] Gorter EA, Krijnen P, Schipper IB (2017). Vitamin D status and adult fracture healing. J Clin Orthop Trauma.

[21] Gatt T, Grech A, Arshad H (2023). The effect of vitamin D supplementation for bone healing in fracture patients: a systematic review. Adv Orthop.

